# SUBJECTIVE AGE AND COGNITIVE, PHYSICAL, AND PSYCHOSOCIAL HEALTH IN OLDER ADULTS

**DOI:** 10.1093/geroni/igz038.2422

**Published:** 2019-11-08

**Authors:** Briana N Sprague, Sara B W Troutman, Sara E Bickhart, Nathanael Jiya, Lesley A Ross

**Affiliations:** 1 The Pennsylvania State University, University Park, Pennsylvania, United States; 2 The Pennsylvania State University, The Pennsylvania State University, United States

## Abstract

Subjective age predicts cognitive, physical, and psychosocial function in older adults independent of objective age. However, the stability of this association is unknown. The goals of this study were to determine whether (1) subjective age was stable across eight years and (2) if subjective age predicted cognitive, physical, and psychosocial function in older adults. This study used data from a nationally-representative longitudinal cohort study (Health and Retirement Study) of 3,084 older adults aged 65-96 measured biannually from 2008 – 2016 with at least two waves of data. The main predictor was the proportion of subjective age compared to one’s objective age. The outcomes were dementia status (TICSm), physical function (grip strength, walk speed), and psychosocial function (Self-Perceptions of Aging). On average, the sample reported feeling 15% younger than their objective age. Among older adults who reported feeling older than the sample average, there were significant deficits in TICSm, walk speed, and self-perceptions of aging, but not in grip strength. Older adults who feel older than average have poorer cognitive, lower limb physical, and psychosocial function, and these deficits are exacerbated when those individuals feel even older compared to their own average. These results suggest that instability in subjective aging is particularly disruptive for those already at risk for poor aging, so interventions targeting the most vulnerable could promote healthy aging.

